# Investigation of Electrical Properties and Reliability of GaN-Based Micro-LEDs

**DOI:** 10.3390/nano10040689

**Published:** 2020-04-06

**Authors:** Ke Zhang, Yibo Liu, Hoi-sing Kwok, Zhaojun Liu

**Affiliations:** 1 Department of Electrical and Electronic Engineering, The Southern University of Science and Technology, Shenzhen 518000, China; kzhangao@connect.ust.hk (K.Z.); yliufl@connect.ust.hk (Y.L.); eekwok@ust.hk (H.-s.K.); 2 Department of Electronic and Computer Engineering, Hong Kong University of Science and Technology, Hong Kong SAR 999077, China

**Keywords:** GaN-based micro-LEDs, reliability test, micro-LED display

## Abstract

In this paper, we report high-performance Micro-LEDs on sapphire substrates, with pixel size scaling to 20 µm and an ultra-high current density of 9902 A/cm^2^. The forward voltages (V_F_) of the devices ranged from 2.32 V to 2.39 V under an injection current density of 10 A/cm^2^. The size and structure-dependent effects were subsequently investigated to optimize the device design. The reliability of Micro-LED devices was evaluated under long-aging, high-temperature, and high-humidity conditions. It was found that Micro-LED devices can maintain comparable performance with an emission wavelength of about 445 nm and a full width at half maximum (FWHM) of 22 nm under extreme environments. Following this, specific analysis with four detailed factors of forward voltage, forward current, slope, and leakage current was carried out in order to show the influence of the different environments on different aspects of the devices.

## 1. Introduction

Micro-LED devices have received great attention recently, owing to their superior properties such as self-emission, high brightness, low power consumption, fast response time and long lifetime [1,2,3,4,5]. With the developments of semiconductor technology and the maturity of miniaturization technology, more and more new applications of III–nitride light-emitting diodes (LEDs) have been realized, such as lighting sources [6], visible light communication (VLC) [7], high-power devices [8] and biomedical devices [9]. One of the hottest topics in recent years is micro-LED displays, which are even expected to become the next generation of displays, replacing conventional display technologies like liquid crystal display (LCD) and organic light emitting diode (OLED). More advanced technology focuses on integrating other functions like sensing, photodetection and solar cells into micro-LED displays, producing fully integrated and multifunctional devices [10,11]. Due to the diversity of their applications, the performance and reliability of Micro-LED devices under some extreme environments have become increasingly important to achieve. Many research works have already made efforts to optimize these devices. In some research into Micro-LEDs, the pixel size was quite large, with a pixel pitch of hundreds of micrometers, and a current density of only several tens of A/cm^2^_,_ due to immature technology [12,13]. Micro-LEDs grown on Si substrate were once regarded as mainstream, due to the compatibility with the existing manufacturing process. However, they often suffered from low efficiency [14,15]. There has also been much work to improve the light extraction efficiency (LEE) of the devices and display panels [16,17,18] in order to achieve better external quantum efficiency (EQE). Park et al., from Korea University, reported using indium tin oxide (ITO) as the electrode to form a transparent device, yet it still led to a low current density [19].

In this work, we designed and fabricate micro-LEDs and arrays on sapphire substrates of different sizes and structures, with the smallest pixel being only 20 µm. The devices showed good performance, with a current density of 9902 A/cm^2^. The relatively small forward voltage (V_F_) of about 2.4 V was achieved under 10 A/cm^2^, which is sufficient for displays. It was also found that the electrical characteristics showed an obvious correlation with the device size. Accordingly, the size-dependent and structure-dependent effects were systematically investigated to show the optimized design. The devices were tested under several extreme environments, including room conditions without packaging for 369 days, a high-temperature condition of 180 °C, and a high-humidity condition of 85 °C and 85% humidity for 48 h. Concerning the performance, the specific factors of forward voltage, forward current, slope and leakage current were separately characterized in order to show the stability and reliability of micro-LED devices under different operation conditions.

## 2. Materials and Methods 

**Figure 1:**
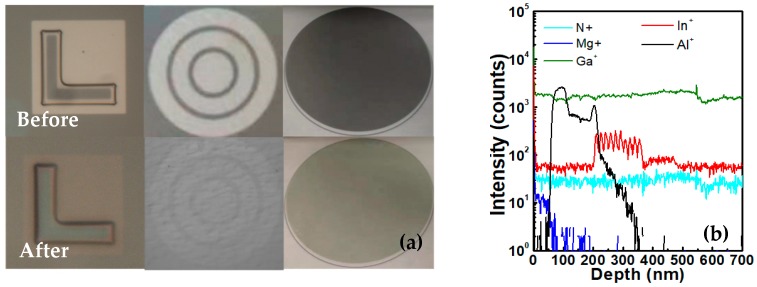
(**a**) Comparison between the samples before and after annealing. (**b**) Composition of the epitaxy measured by TOF-SIMs.

The micro-LED devices were fabricated with commercially available LED wafers. A time-of-flight secondary ion mass spectrometer (TOF-SIMS, Hong Kong, China) was used to analyze the composition of the epitaxial wafer, as shown in Figure 1b. The 3000 Å SiO_2_ was grown by Plasma Enhanced Chemical Vapor Deposition (PECVD) as a hard mask for following dry etching. This is because serious deformation was observed in the corner of very small pixels, such as 10 µm pixels, if only photoresist (PR) was used as mask. Photolithography and buffered oxide etch (BOE) wet etching were adopted to pattern SiO_2_. To enhance the adhesion of PR on the SiO_2_, hexamethyldisilazane (HDMS) and extended soft baking and post baking of 110 °C for 4 min were performed. The mesa structure was achieved by a GaN etcher with a gas of 25 sccm Cl_2_ and 5 sccm BCl_3_. The etching procession was divided into four cycles and the final depth was 0.7 µm. Surface treatment was performed subsequently to clean the samples and reduce plasma damage. The current spreading layer, consisting of 40/40Å Ni/Au, was evaporated to increase the current uniformity with the optimized thickness to reduce the light absorption. Following this, an annealing process was performed for 5 mins at 570 °C in an atmosphere of N_2_:O_2_ = 4:1 in order to enhance the transparency as shown in Figure 1a. Through comparison of the samples before and after annealing (Figure 1a), it is clear that the transparency has been improved. The p and n electrodes were made of evaporated stacks of Ti/Al/Ni/Au with 300 Å/700 Å/1000 Å/500 Å. Titanium was used to enhance the adhesion between CSL and aluminum. Thick aluminum and nickel can improve the horizontal current uniformity. Gold was adopted to avoid the electrode being oxidized.

## 3. Results and Discussion

**Figure 2:**
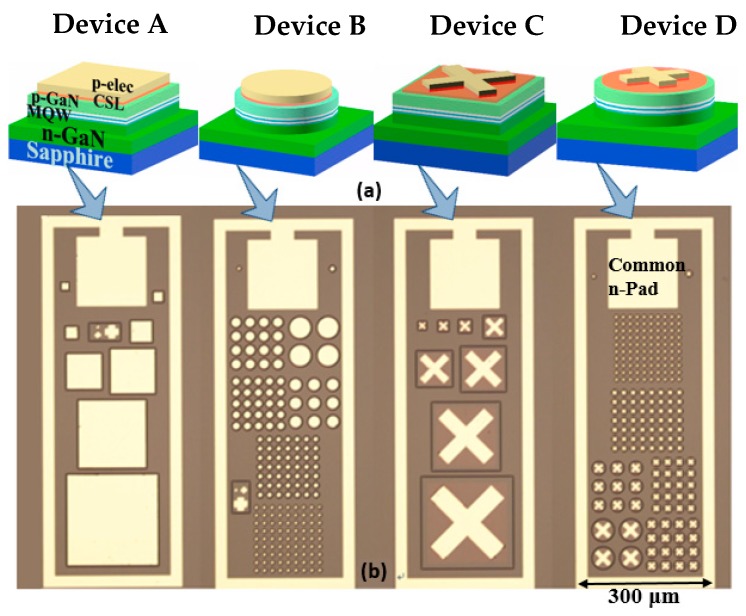
(**a**) Three-dimensional views of micro-LEDs; (**b**) microscope photos of the device dies of different sizes and structures (from left to right: bottom emitting single pixels, bottom emitting pixel array, top emitting single pixels and top emitting pixel array).

Micro-LEDs of varying sizes from 200 µm to 20 µm were designed and fabricated. To further investigate the electrical designs, mesa designs, and emitting configurations, four types of devices were studied: bottom emitting pixel with a square shape (Device A), bottom emitting pixel with a circular shape (Device B), top emitting pixel with a square shape (Device C), and top emitting pixel with a circular shape (Device D). The detailed three-dimensional views of each of the four types of devices, and the optical microscope images of the micro-LED dies of different sizes and structures, are shown in Figure 2. In order to investigate the relationship between device structure and performance, the pixel sizes of A–D structures were all fixed as 50 µm. Therefore, Device A had a 50 µm mesa length and 40 µm electrode length, Device B had a 50 µm mesa diameter and 40 µm electrode diameter, Device C was designed with a 50 µm mesa length and a 40 µm cross electrode diameter, and Device D had a 50 µm mesa diameter and a 40 µm cross electrode diameter.

### 3.1. Structure dependent effect

**Figure 3:**
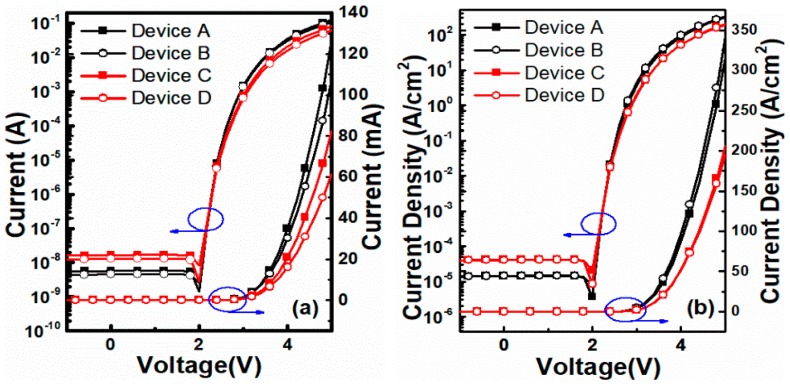
(**a**) *I-V* and (**b**) *J-V* characteristics of micro-LEDs of 4 types of structures.

Four types of micro-LED structures were designed to investigate the relationship between structures and performance. In Figure 3a, at an applied voltage of 5 V, the operation current of Device A is the largest and is two times that of Device D. From Figure 3b, it is noticed that the geometric shape of micro-LEDs has little effect on the current density. Bottom emitting devices have superior performance, with a 60% higher current density compared to top emitting devices. In the log scale, the leakage current is strongly affected by the emitting structure but not the geometric shape. The leakage current of the bottom emitting structure is 2.6 nA, while that of the top structure is three times larger. Therefore, Device A is the best choice, with a higher forward current and forward current density, and a lower leakage current, and it will be used in future research.

### 3.2. Size dependent effect

**Figure 4:**
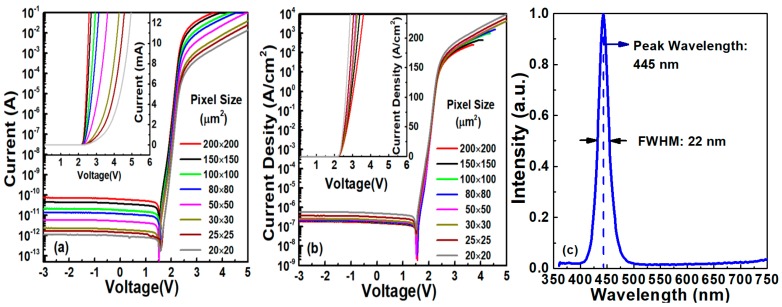
(**a**) *I-V*, (**b**) *J-V* characteristics of micro-LED devices with different pixel sizes, (**c**) EL spectrum.

Figure 4a,b shows the representative *I-V* and *J-V* characteristics, respectively, of the micro-LED with a bottom emitting and square mesa structure (Device A). The slope of the *I-V* characteristics in the log scale is about 110 mV/dec and the leakage current ranges from 1 pA to 80 pA. V_F_ can be as small as 2.36 V at 10 A/cm^2^, which is sufficient for display applications, and the deviation is less than 3%. The forward current has a positive correlation with pixel size at the same V_F_, which is consistent with the Shockley diode equation. It can be noticed that, as the pixel size scales, a higher current density can be achieved, which is tentatively attributed to the better current spreading phenomenon [20]. In the electroluminescence spectrum (Figure 4c), the micro-LED devices had a peak wavelength of 445 nm, and the FWHM is 22 nm. The average luminous efficacy of micro-LED devises with pixel size from 50 µm to 200 µm was 25.6 lm/W, with a deviation of ±5%, which is 82.98% of CIE standard.

### 3.3. Reliability and Stability

Though micro-LEDs are praised for their long lifetimes, it has been found that a small amount of degradation also occurs in extreme environments, which may cause problems for multifunctional applications such as VLC. The devices, without any packaging, were tested under different conditions in order to investigate their reliability and stability. The degradation factors in terms of forward current, forward voltage, slope and leakage current were summarized and compared. Here, slope (*S*) is defined as the reciprocal of the slope in the *I-V* curves with a unit of mV/dec, which is important for VLC and sensor applications. 

**Figure 5:**
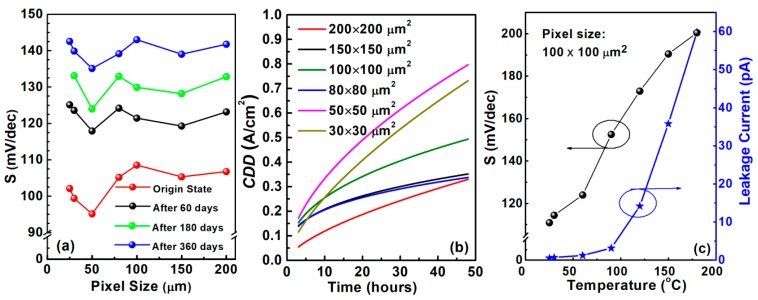
(**a**) Slope degradation trend of micro-LEDs as aging increases; (**b**) current density deviation trend at 3 V at 85 °C and 85% relative humility; (**c**) *S* and leakage current performance under different temperatures.

In Figure 5a, it is obvious that micro-LEDs of different sizes have similar S degradation trends. The slope degradation rate (SDR) is defined in Equation (1). After fitting analysis, it was found that the SDRs were quite high in the first 60 days and later reduce exponentially. After 150–200 days, the SDRs gradually reached a stable state, 2–5 times lower than the original SDRs. The stabilized degradation rate is 0.13–0.37 mv/(dec·day), showing sufficient stability over a long lifetime.

(1) $$\mathrm{SDR}=\frac{Safter-Soriginal}{taging\;time}$$

Micro-LEDs also have good humidity stability, especially compared to organic material. It is reported that OLEDs degrade significantly without encapsulation and degrade by half with basic encapsulation within 20 h [21]. Micro-LEDs without any packaging can maintain almost the same performance over 48 h under the same conditions. Forward currents at 3 V and V_F_ without packaging degraded less than 0.15 mA and 0.25 V, respectively, after 48 h under the conditions of 85 °C and 85% relative humility. Figure 5b is the current density deviation fitting curve. Pixels of varying sizes show a similar trend, but smaller pixels tended to degrade faster.

To investigate the temperature impact on micro-LEDs, the devices were characterized under temperature conditions ranging from room temperature (RT) to 180 °C, as shown in Figure 5c. It is noted that the slope has risen 81.8% to 200 mV/dec. The leakage currents also show a temperature dependence effect, increasing from 1 pA to 180 pA at a reverse voltage of 6 V; however, it is still small enough to be ignored for device operation. Therefore, micro-LEDs of different sizes can function properly under high-temperature conditions.

**Figure 6:**
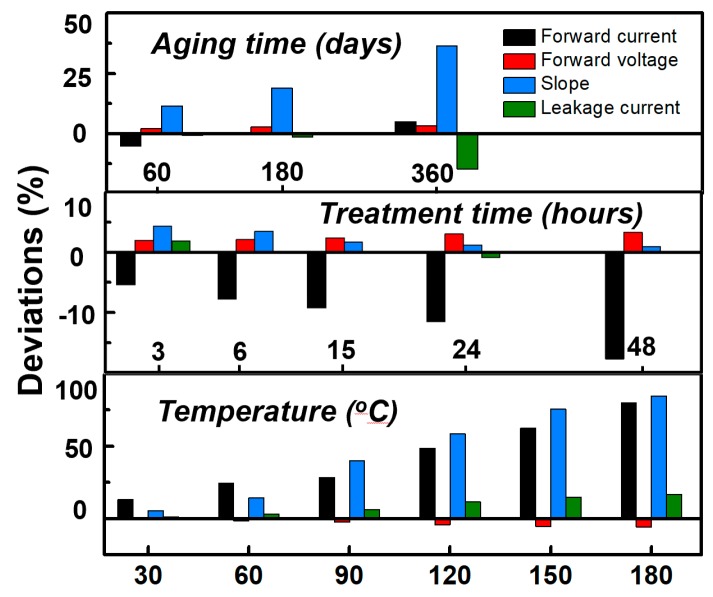
The degradation comparisons of forward current, forward voltage, slope and leakage current under long aging time, high-humidity treatment, and high-temperature conditions.

The degradations of forward current, forward voltage, slope and leakage current under different conditions are summarized and compared, as shown in Figure 6 and Table 1. The forward current and forward voltage were fixed as 3 V and 10 A/cm^2^, respectively. The deviation of the leakage current was compared after processing by log function, due to the leakage being too small to show the trend directly.

The long aging time test was carried out in a common room environment for about one year. The forward current and voltage change were small enough to be ignored, demonstrating the long operation lifetime properties of the device. The leakage current slightly decreased over the year as well, while the slope factor increased 45%. This means that if the device works as a sensor or has a communication function, the sensitivity may slightly decrease with aging. Therefore, proper packaging must be taken into consideration for these kinds of functions. Under the high-humidity test of 85 °C and 85% conditions, the forward current gradually decreased more than 15%; correspondingly, the forward voltage and slope increased and decreased, respectively. The leakage current was not affected by the humidity, almost remaining the same. The deviation was most significant under the temperature test from room temperature to 180 °C. Both the forward current and leakage current had an obvious rise. This may have been caused by the change in the Fermi distribution function, which is related to the temperature. As a result, the carrier concentration increased. In Figure 6, the leakage current increase is about 25%, and the forward current and the slope have the largest increase at more than 70%.

The performance of each of our devices is compared with those from other groups [22,23,24,25,26,27,28,29] in Table 2. The applied bias of the devices is 5 V, which is typically reported. It can be seen that the devices of different sizes reported in this paper have better electrical properties than the reported devices.

## 4. Conclusions

Four types of micro-LED structures were designed and analyzed, including bottom emitting pixel with a square shape, bottom emitting pixel with a circular shape, top emitting pixel with a square shape, and top emitting pixel with a circular shape. Through the analysis of the *I-V* and *J-V* characteristics, it was found the leakage current is strongly affected by the emitting structure but not the geometric shape. The geometric shape also has an influence on the current uniformity of the devices. Additionally, the leakage current is strongly affected by the size of the emitting structure. As a result, the bottom emitting pixel with a square shape is the best choice and will be adopted in future research. To investigate the size dependence, micro-LEDs with pixel size ranges from 20 µm to 200µm were fabricated. It was found that a higher current density can be achieved as the pixel size scales down. Micro-LEDs and arrays with different structures and sizes were designed and fabricated. A relatively high current density of 9902 A/cm^2^ under 5 V forward bias was achieved. The V_F_ was reduced to less than 2.4 V when the current density was fixed as 10 A/cm^2^, which is enough for display applications and the corresponding typical V_F_ is usually larger than 3 V. Three degradations of micro-LEDs under extreme environments were subsequently investigated, including long aging time, high-humidity treatment and high-temperature environment. It was found that under long aging in a room environment for 1 year, the device was able to maintain almost the same performance, except for the slope factor, with a 45% raise, which is important for sensitive applications such as sensors and VLC. Under moist conditions over a period of 48 h, the operation currents decreased 15% and the leakage current increased little. Temperature was the most significant factor and clearly affected devices in operation current and voltage, S performance and leakage current. The leakage current increased about 25%, and the forward current and the slope had the largest increases at more than 70%. The device can still work properly at 180^°^C, showing good reliability of the device. To optimize the micro-LED devices and improve the efficiency, further heat dissipation should be one of the most important topics to investigate in the future.

## Figures and Tables

**Table 1:** Reliability test results of micro-LED devices under different conditions.

	Condition	I (mA) @ 3V	V_F_ (V) @ 10A/cm^2^	S (mV/dec)	leakage (A) @ −5V
Aging (days)	0	13	2.43	108.5	5.3E−13
	60	12	2.48	121	4.3E−13
	180	13	2.50	129	3.7E−13
	360	14	2.51	148	8.4E−15
Temperature (°C)	25	13	2.43	108.5	5.3E−13
	30	17	2.42	114.4	7.1E−13
	60	17	2.39	124	1.2E−12
	90	17	2.37	152	3.2E−12
	120	19	2.33	172	1.4E−11
	150	21	2.3	190.4	3.6E−11
	180	23	2.29	200.4	6.0E−11
Humidity (hours)	0	14	2.43	108.6	5.2E−13
	3	12	2.48	113.2	8.7E−13
	6	13	2.48	116.2	5.0E−13
	15	12	2.49	110.3	5.5E−13
	24	12	2.50	109.8	4.1E−13
	48	11	2.51	109.6	5.4E−13

**Table 2:** Current density comparison under a 5V forward bias (units: A/cm^2^).

Pixel Size (µm)	This Work	University of Strathclyde	Korea University	HKUST	Fudan University	LETI
20	9902	-	-	-	-	70 [29]
25	6338	1500 [13]	-	-	1750 [27]	-
30	4700	-	200 [22]	-	-	-
50	4400	-	4000 [23]	825 [24]	1000 [27]	30 [29]
80	2077	1400 [13]	-	918 [25]	937 [28]	-
100	1700	150 [14]	-	90 [26]	712 [28]	-
